# Statins do not prevent acute organ failure in ventilated ICU patients: single-centre retrospective cohort study

**DOI:** 10.1186/cc10063

**Published:** 2011-02-28

**Authors:** Marius J Terblanche, Ruxandra Pinto, Craig Whiteley, Stephen Brett, Richard Beale, Neill KJ Adhikari

**Affiliations:** 1 Critical Care & Anaesthesia Research Group, King's College London, St Thomas' Hospital, Westminster Bridge Road, London SE1 7EH, UK; 2 Department of Critical Care Medicine, Guy's & St Thomas' NHS Foundation Trust, London SE1 7EH, UK; 3 Department of Critical Care Medicine and Sunnybrook Research Institute, Sunnybrook Health Sciences Centre, and University of Toronto, 2075 Bayview Avenue, Toronto, Ontario M4N 3M5, Canada; 4 Centre for Perioperative Medicine and Critical Care Research, Imperial Health Care NHS Trust, Hammersmith Hospital, Du Cane Road, London W12 0HS, UK

## Abstract

**Introduction:**

Observational studies suggest statin therapy reduces incident sepsis, but few studies have examined the impact on new organ failure. We tested the hypothesis that statin therapy, administered for standard clinical indications to ventilated intensive care unit patients, prevents acute organ failure without harming the liver.

**Methods:**

We performed a retrospective, single-centre cohort study in a tertiary mixed medical/surgical intensive care unit. Mechanically ventilated patients without nonrespiratory organ failure within 24 hours after admission were assessed (during the first 15 days) for new acute organ failure (defined as Sequential Organ Failure Assessment (SOFA) score 3 or 4), liver failure (defined as new hepatic SOFA ≥3, or a 1.5 times increase of bilirubin from baseline to a value ≥20 mmol/l), and alanine transferase (ALT) > 165 IU/l. The effect of statin administration was explored in generalised linear mixed models.

**Results:**

A total of 1,397 patients were included. Two hundred and nineteen patients received a median (interquartile range) of three (two, eight) statin doses. Patients receiving statins were older (67.4 vs. 55.5 years, *P *< 0.0001), less likely female (25.1% vs. 37.9%, *P *= 0.0003) and sicker (Acute Physiology and Chronic Health Evaluation (APACHE) II score 20.3 vs. 17.8, *P *< 0.0001). Considering outcome events at least 1 day after statin administration, statin patients were equally likely to develop acute organ failure (28.4% vs. 22.3%, *P *= 0.29) and hepatic failure (9.5% vs. 7.6%, *P *= 0.34), but were more likely to experience an ALT increase to > 165 IU/l ((11.2% vs. 4.8%, *P *= 0.0005). Multivariable analysis showed that APACHE II score (odds ratio (OR) = 1.05 per point; 95% confidence interval (CI) = 1.03 to 1.07) and APACHE II admission category (*P *< 0.0001), but not statin administration (OR = 1.21; 95% CI = 0.92 to 1.62), were significantly associated with acute organ failure occurring on or after the day of first statin administration. Statin administration was not associated with liver impairment (OR = 1.08; 95% CI = 0.66 to 1.77) but was associated with a rise in ALT > 165 IU/l (OR = 2.25; 95% CI = 1.32 to 3.84), along with APACHE II score (*P *= 0.016) and admission ALT (*P *= 0.0001).

**Conclusions:**

Concurrent statin therapy does not appear to protect against the development of new acute organ failure in critically ill, ventilated patients. The lack of effect may be due to residual confounding, a relatively low number of doses received, or an absence of true effect. Randomised controlled trials are needed to confirm a protective effect.

## Introduction

Many patients suffering from severe infections and early sepsis - conditions associated with deterioration and the development of acute organ failure - require mechanical ventilation after intensive care unit (ICU) admission [1-3]. Mechanical ventilation is associated with ventilator-associated pneumonia and increases the risk of developing other nonrespiratory organ failures [1]. In this context, acute organ failure appears to occur mostly during the first 10 days after admission and is associated with an increased risk of death [1,3,4]. Because many mediator-targeting treatments for established severe sepsis have failed in randomised trials, an incentive exists to prevent the onset of acute organ failure [5].

A candidate therapy to prevent acute organ failure is the statin class of drugs, which may dampen the disproportionate innate immune response following microbial invasion [6-10]. While numerous observational studies support the notion of improved sepsis-related outcome in those patients receiving long-term statin therapy, it is not known whether such therapy protects critically ill patients against the development of new acute organ failure or the worsening of existing organ dysfunction [11,12]. Furthermore, while statins are generally safe and well tolerated in the outpatient population, their safety in the critically ill patient, particularly with respect to liver function, is unknown [13].

Our objectives were to determine whether statin therapy, as administered by clinicians to a cohort of mechanically ventilated patients without nonrespiratory organ failure within 24 hours of ICU admission, (1) reduces the incidence of a composite endpoint of new nonrespiratory organ failure or the worsening of existing respiratory dysfunction during the first 15 days of admission, and (2) is associated with liver impairment as determined by changes in bilirubin and alanine transferase (ALT). We hypothesised that statin therapy does not worsen liver function in this high-risk, clinically important population and protects them against new acute organ failure.

## Materials and methods

### Study design and setting

We performed a retrospective cohort study in which all patients (regardless of diagnosis) receiving mechanical ventilation but without nonrespiratory failure within 24 hours of admission to the ICU were followed until ICU discharge, for a maximum of 15 days [14]. During the follow-up period we assessed organ function (defined by the Sequential Organ Failure Assessment (SOFA) score) and ALT levels daily [14].

The study was performed in a single, tertiary academic, medical-surgical ICU and included patients admitted between June 2002 and May 2006. Data were extracted from the Clinical Information System (CareVue™; Philips, Amsterdam, The Netherlands). The Clinical Information System is used for all aspects of patient management. All drug prescriptions are electronic, and the system interfaces with the institution's laboratory database and all monitoring equipment and thus stores all physiological, treatment and pharmacological information related to the patient's ICU stay.

### Participants

All adult (> 16 years of age) patients requiring mechanical ventilation on admission to the ICU were eligible. We excluded those patients re-admitted to the ICU during their current hospitalisation, patients with any nonrespiratory organ failure within 24 hours of admission (defined by SOFA ≥3), and patients missing admission data that precluded the determination of baseline organ function.

### Data collection and follow-up

Raw data were extracted from the Clinical Information System and transferred to a relational database (Microsoft Access™; Microsoft, Seattle, WA, USA). The study database was programmed to calculate the SOFA score.

Patients were followed until ICU discharge, for a maximum of 15 days, because previous data from a large international study suggest that deterioration occurs mostly during the first 10 days after admission [1]. During the follow-up period, physiological, biochemical and treatment data were collected daily. The vital status at ICU discharge and hospital discharge and the respective lengths of stay were recorded.

### Exposure and outcome definitions

The main exposure variable was statin therapy received in the ICU during the follow-up period. Statin exposure was defined as the documented administration of a prescribed dose at any time during the 15-day follow-up period.

The specific indications for the statin prescription were unknown. The critical care pharmacists, however, routinely contacted the patients' healthcare providers and families to elicit information regarding chronic medications, including prior statin prescriptions. During the study period it was ICU policy, monitored by the critical care pharmacists, to continue statin therapy when previously prescribed and to start a new prescription for recognised indications (for example, an acute coronary syndrome).

There were three outcomes of interest. Acute organ failure was defined by either worsening *respiratory *function compared with admission (defined as achieving a SOFA respiratory score of 3 or 4 in those with a lower score (0, 1 or 2) on admission, or an increase in SOFA respiratory score to 4 for those with a baseline SOFA of 3) or new *nonrespiratory *organ failure (defined by a SOFA score of 3 or 4 for any of the cardiovascular, renal, hepatic, or haematological systems). Since all patients were sedated we did not consider the neurological element of SOFA in the analysis. The outcome of *liver impairment *was defined either by new hepatic failure, (defined as hepatic SOFA ≥3) or by an increase of bilirubin ≥1.5 times from baseline to a value ≥20 mmol/l. We separately considered the outcome of maximum ALT > 165 IU/l (three times the laboratory's upper limit of normal).

We were interested in measuring the effect of statin administration on both the incidence of liver failure (as defined by SOFA) and on more subtle changes in function. We therefore deliberately used conservative cut-off values to increase sensitivity and avoid bias away from the null hypothesis of no harm.

### Statistical methods

Data are presented as the mean (standard deviation), median (interquartile range) or number (percentage) as appropriate. Baseline differences between exposure groups were compared using Student's *t *test or the Wilcoxon rank sum test for continuous variables and the chi-square test or Fisher's exact test for binary variables, as appropriate. We report the proportion of patients with missing values. In unadjusted analyses, we handled missing data in the following manner. Values of bilirubin missing on some days were imputed on a value carried forward or value carried backward basis. Bilirubin (*n *= 20) and ALT (*n *= 21) values missing on all patient-days were assumed to be normal, representing the most conservative approach. To calculate the number of days of acute organ failure, liver impairment, and ALT > 165 IU/l, we counted the number of days that each of these events occurred without requiring that they be consecutive.

Statin patients were classified as unexposed during the days preceding the administration of the first statin dose, and as exposed thereafter. For the descriptive analyses, we report for the statin group the number of outcome events that occurred before statin administration, on the same day or after statin administration, and at least 1 day after statin administration.

To investigate the effect of treatment duration we performed *post hoc *analyses, comparing the outcome events in patients who received at least seven statin doses with nonstatin-exposed patients who were in the ICU for at least 7 days.

We analysed the effect of statin administration on the outcomes of interest using a generalised linear mixed model with logit link function while accounting for repeated measures using an autoregressive correlation structure. Odds ratios (OR) with 95% confidence intervals (CIs) are presented. The effect of statins was adjusted for age, gender, admission APACHE II score, baseline total SOFA score (excluding the neurological component), and main admission category (as defined by APACHE II score) [15]. For the generalised linear mixed models we considered outcome events in the statin group if they occurred on the day of statin or after statin administration.

We interpreted *P *≤ 0.05 as statistically significant using two-sided tests. We used SAS version 9.2 software (SAS Institute, Cary, NC, USA) for all analyses. We used a convenience sample size based on the data available for the chosen study period to calculate effect estimates and confidence intervals.

### Ethics

The present study was approved by the institutional review board of Guy's & St Thomas' NHS Foundation Trust, which waived the need for informed consent.

## Results

### Descriptive data

**Figure 1:**
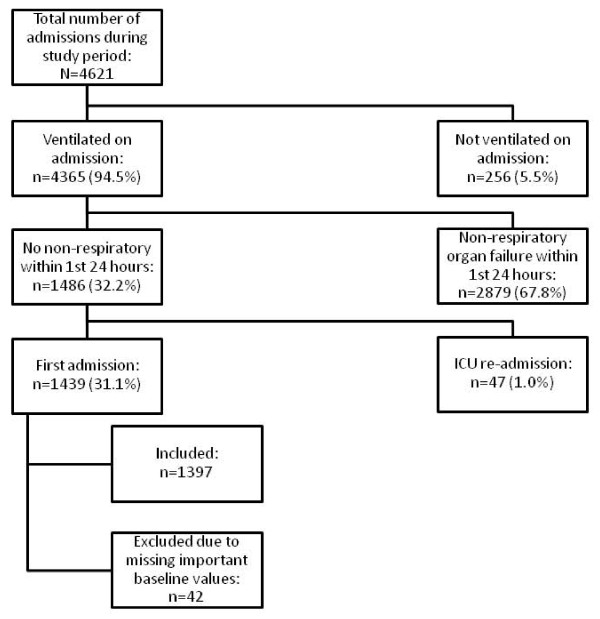
**Flow diagram of patients meeting the eligibility criteria**. ICU, intensive care unit.

During the study period, 4,621 patients were admitted to the ICU (see Figure 1). Of these, 3,135 patients were excluded because they were not ventilated, had nonrespiratory organ failure, had already been admitted to the ICU during the same hospitalisation or had missing baseline data. We therefore included 1,397 patients in the final study cohort. The admission category, as defined by APACHE II score, was missing in 45/1,397 (3.2%) patients while 17/1,397 (1.26%) patients were categorised as metabolic. Because only one patient in the metabolic category received a statin, the entire category was excluded from the regression analyses.

Two hundred and nineteen patients received a median of 3 (2, 8) statin doses (Table 1). Of these, 70 patients (32.0%), 77 patients (35.2%), 27 patients (12.3%) and 17 patients (7.8%) were started on days 1, 2, 3 and 4, respectively. The most common statin administered was simvastatin (72.2%; median dose 20 mg, range 10 to 80 mg), followed by atorvastatin (20.9%) and pravastatin (6.9%).

**Table 1:** Baseline characteristics

Variable	Overall (*n *= 1,397)	No statin exposure (*n *= 1,178)	Statin exposed (*n *= 219)	*P *value
Age (years)	57.4 (18.4)	55.5 (18.9)	67.4 (10.3)	< 0.0001
APACHE II score	18.2 (6.8)	17.8 (6.9)^a^	20.3 (6.0)	< 0.0001
Female	501 (35.9%)	446 (37.9%)	55 (25.11%)	0.0003
Reason for admission^b^				< 0.0001
Infection	320 (22.9%)	269 (22.9%)	51 (23.3%)	
Sepsis/septic shock	44 (3.2%)	39 (3.3%)	5 (2.3%)	
Cardiac failure	31 (2.2%)	16 (1.4%)	15 (6.9%)	
Haemorrhage	48 (3.4%)	46 (3.9%)	2 (0.9%)	
Postoperative ventilation (planned)	176 (12.6%)	147 (12.5%)	29 (13.2%)	
Postoperative ventilation (unplanned)	149 (10.7%)	112 (9.5%)	37 (16.9%)	
Other^c^	628 (45.0%)	548 (46.6%)	80 (36.5%)	
Number of statin doses received			3 (2, 8)	
Main admission category^d^				< 0.0001
Cardiovascular	464 (34.3%)	347 (30.5%)	117 (54.4%)	
Gastrointestinal	118 (8.7%)	110 (9.7%)	8 (3.7%)	
Metabolic	17 (1.3%)	16 (1.4%)	1 (0.5%)	
Neurological	302 (22.3%)	286 (25.2%)	16 (7.4%)	
Respiratory	451 (33.4%)	378 (33.2%)	73 (33.95%)	
Admission source				< 0.0001
Emergency room	369 (26.4%)	326 (27.7%)	43 (19.6%)	
Hospital ward	221 (15.8%)	175 (14.9%)	46 (21%)	
Another hospital	294(21.05%)	263 (22.3%)	31 (14.2%)	
Operating room	377(26.99%)	310 (26.3%)	67 (30.6%)	
High dependency ward	125 (8.95%)	98 (8.3%)	27 (12.3%)	
Other	11 (0.79%)	6 (0.51%)	5 (2.3%)	
Baseline biochemistry				
Bilirubin (mmol/l)	17 (12, 25)	17 (12, 26)^e^	17 (12, 23)	0.31
ALT (IU/l)	25 (15, 48.5)	24 (15, 46)^f^	34 (18, 64)	< 0.0001
Baseline SOFA				
Total	3 (2, 4)	3 (2, 4)	4 (3, 5)	< 0.0001
Median total nonrespiratory	1 (0, 2)	1 (0, 2)	1 (0, 2)	0.0005
Mean total nonrespiratory	1.23 ± 1.29	1.18 ± 1.28	1.49 ± 1.31	0.001
Respiratory:				< 0.0001
0	70 (5.01%)	62 (5.3%)	8 (3.6%)	
1 or 2	973 (69.7%)	843 (71.6%)	130 (59.4%)	
3 or 4	354 (25.3%)	273 (23.2%)	81 (37.0%)	
Hepatic 1 or 2	591 (42.3%)	503 (42.7%)	88 (40.2%)	0.49
Renal 1 or 2	328 (23.5%)	243 (20.6%)	85 (38.8%)	< 0.0001
Haematological 1 or 2	341 (24.4%)	284 (24.1%)	57 (26.0%)	0.54
Cardiovascular 1 or 2	28 (2%)	14 (1.2%)	14 (6.4%)	< 0.0001

Data expressed as mean ± standard deviation, median (interquartile range), or number (percentage). ALT, alanine transferase; APACHE, Acute Physiology and Chronic Health Evaluation; SOFA, Sequential Organ Failure Assessment. ^a^Data missing for 16 patients. ^b^Data missing for one patient. ^c^Include trauma (*n *= 44, 3.2%), overdose (*n *= 96, 6.9%), seizures (*n *= 84, 6.0%), post-arrest (*n *= 117, 8.4%) and abdominal obstruction or perforation (*n *= 54, 3.9%). ^d^Data missing for 41 patients in the nonstatin group and four patients in the statin group. ^e^Data missing for 20 patients. ^f^Data missing for 21 patients.

Patients receiving statins were older (67.4 vs. 55.5 years, *P *< 0.0001), less likely female (25.1% vs. 37.9%, *P *= 0.0003) and had higher APACHE II scores (20.3 vs. 17.8, *P *< 0.0001) than the nonstatin patients. Admission respiratory function was worse in the statin group (*P *< 0.0001), which had more prevalent respiratory failure (SOFA 3 or 4) and less prevalent respiratory dysfunction (SOFA 1 or 2).

The baseline total nonrespiratory SOFA score was higher in the statin group due to the presence of higher extreme values: median values were 1 (0, 2) for both groups (*P *= 0.0005), but the mean was 1.49 (1.31) in the statin group and 1.18 (1.28) in the nonstatin group (*P *= 0.001). At baseline, statin patients were 1.9 and 5.3 times more likely to have renal (*P *< 0.0001) and cardiovascular (*P *< 0.0001) failure, respectively, but there was no difference in haematological failure (*P *= 0.54). There was no difference in baseline hepatic SOFA score (*P *= 0.49), although the baseline ALT was statistically significantly higher in the statin group (34 vs. 24 IU/l, *P *< 0.0001).

### Unadjusted outcome data

Overall, 380 (27.2%) patients developed acute organ failure. Of these patients, 21 (1.5%) in the statin group developed acute organ failure *before *a statin was administered. Sixty-seven (33.8%) statin patients developed acute organ failure on or after the day of first statin administration compared with 292 patients (24.8%) in the nonstatin group (*P *= 0.0073) (Table 2). Furthermore, 52 (28.4%) statin patients developed acute organ failure at least 1 day after the first statin administration compared with 292 (24.8%) nonstatin patients (*P *= 0.29). There were also no differences in the time to organ failure (3 (3, 4) vs. 3 (2, 5) days in statin vs. nonstatin groups; *P *= 0.63) or duration of organ failure (2 (1, 5) days in both groups; *P *= 0.77).

**Table 2:** Unadjusted outcome data

Variable	Overall (*n *= 1397)	Nonstatin group (*n *= 1,178)	Statin group (*n *= 219)	*P *value
Organ failure				
On or after day of first statin administration	359/1,376 (26.1%)	292/1,178 (24.8%)	67/198 (33.8%)	0.007
At least 1 day after first statin administration	344/1,361 (25.3%)	292/1,178 (24.8%)	52/183 (28.4%)	0.29
Days to organ failure	3 (2, 5) (*n *= 359)	3 (2, 5) (*n *= 292)	3 (3, 4) (*n *= 67)	0.63
Duration of organ failure^a^	2 (1, 5) (*n *= 359)	2 (1, 5) (*n *= 292)	2 (1, 5) (*n *= 67)	0.77
Safety				
Hepatic failure^b^	112/1,392 (8.0%)	89/1,178 (7.6%)	23/214 (10.8%)	0.11
Days to hepatic failure	4 (3,8) (*n *= 112)	5 (3, 8) (*n *= 89)	4 (3, 6) (*n *= 23)	0.46
Duration of hepatic failure	1.5 (1, 3) (*n *= 112)	2 (1, 3) (*n *= 89)	1 (1, 4) (*n *= 23)	0.84
ALT > 165 IU/l^c^	77/1,313 (5.9%)	54/1,115 (4.8%)	23/198 (11.6%)	0.0002
Days to ALT > 165 IU/l	7 (4, 11) (*n *= 77)	6 (4, 11) (*n *= 54)	8 (5, 12) (*n *= 23)	0.35
Duration of ALT > 165 IU/l	3 (1, 4) (*n *= 77)	2 (1, 4) (*n *= 54)	3 (1, 4) (*n *= 23)	0.66
Maximum ALT	35 (19, 73) (*n *= 1,292)	33 (18, 69) (*n *= 1,094)	50 (28, 110) (*n *= 198)	< 0.0001
Other outcomes				
ICU mortality	177/1,397 (12.7%)	149/1,178 (12.7%)	28/219 (12.8%)	0.96
Hospital mortality	270/1,397 (19.3%)	221/1,178 (18.8%)	49/219 (22.4%)	0.21
ICU length of stay	5 (3, 10) (*n *= 1,397)	4 (3, 9) (*n *= 1,178)	7 (4, 15) (*n *= 219)	< 0.0001
Hospital length of stay	15 (8, 33) (*n *= 1,397)	14 (7, 31) (*n *= 1,178)	21 (12, 43) (*n *= 219)	< 0.0001

Data expressed as mean ± standard deviation, median (interquartile range), or number (percentage). ALT, alanine transferase; ICU, intensive care unit. ^a^Includes four patients in the statin group who had a Sequential Organ Failure Assessment (SOFA) score (hepatic) ≥3 on the day of first statin administration. ^b^New hepatic failure on the day of or after the first day of statin administration (defined as hepatic SOFA score ≥3) or an increase of bilirubin by ≥1.5 times from baseline to a value ≥20 mmol/l. Missing bilirubin values were assumed to be normal. ^c^Excludes those in statin group with ALT > 165 IU/l at baseline or occurring before the first statin administration. Missing ALT values were assumed to be normal.

In total, 117 (8.4%) patients developed liver impairment. Of these, five patients (0.4%) in the statin group developed liver impairment *before *a statin was administered. There were no differences observed in statin patients who developed liver impairment on or after the first stain administration (10.8% vs. 7.7%, *P *= 0.11), or in statin patients who developed liver impairment at least 1 day after the first statin administration (9.5% vs. 7.6%, *P *= 0.34). Again, no differences were seen in the time to liver impairment (4 (3, 6) vs. 5 (3, 8) days; *P *= 0.46) or duration of liver impairment (1 (1, 4) vs. 2 (1, 3) days; *P *= 0.84).

At baseline, 84 patients had an ALT above the *a priori *defined value of 165 IU/l that defined an outcome, and were therefore not included in the ALT analysis. In the remainder, the maximum ALT was 33 (18, 69) and 50 (28, 110) IU/l in the nonstatin and statin groups, respectively. The ALT rose above 165 IU/l in 77 (5.9%) patients, and occurred more frequently in statin patients (11.6% considering events on or after the day of first statin administration vs. 4.8%, *P *= 0.0002). Similarly, a rise in ALT above 165 IU/l was more common in the statin group when outcomes at least 1 day *after *the first statin administration were considered (11.2% vs. 4.8%, *P *= 0.0005). The timing of onset (8 (5, 12) vs. 6 (4, 11) days after ICU admission, *P *= 0.35) and the duration of the ALT rise (3 (1, 4) vs. 2 (1, 4) days, *P *= 0.66) were similar in statin and nonstatin patients.

The overall lengths of ICU and hospital stays were 5 (3, 10) and 15 (8, 33) days, respectively. Patients receiving statins stayed longer in the ICU (by 3 days, *P *< 0.0001) and in the hospital (by 7 days, *P *< 0.0001). Overall mortality in the ICU (12.7%) and hospital (19.3%) was similar in statin and nonstatin patients (*P *= 0.96 and *P *= 0.21, respectively).

### Regression analyses

In univariable analysis, statin exposure, increasing age, higher admission APACHE II and admission SOFA scores, and APACHE II admission category were associated with acute organ failure (Table 3). After covariate adjustment, the effect of statin administration was nonsignificant (OR = 1.22; 95% CI = 0.92 to 1.62; *P *= 0.17); only the APACHE II score (OR = 1.05 per point; 95% CI = 1.03 to 1.07; *P <*0.0001) and APACHE II admission category (*P *< 0.0001) were significantly associated with acute organ failure. Relative to the APACHE II respiratory admission category, the cardiovascular category was associated with a higher risk of acute organ failure (OR = 1.34; 95% CI = 1.06 to 1.69; *P *= 0.015), while the neurological category was associated with a lower risk (OR = 0.48; 95% CI = 0.33 to 0.71; *P *= 0.0002). Duration of treatment of at least 7 days was not associated with acute organ failure (*n *= 437; OR = 0.81; 95% CI = 0.53 to 1.23; *P *= 0.33).

**Table 3:** Predictors of acute organ failure occurring on or after the first day of statin administration

**Variable (*n *= 1,319)**^**a**^	Univariable analyses			Multivariable analyses		
		
	Odds ratio	95% CI	*P *value	Odds ratio	95% CI	*P *value
Statin versus no statin	1.40	1.06 to 1.84	0.016	1.22	0.92 to 1.62	0.17
Age^b^	1.008	1.00 to 1.01	0.007	1.00	0.99 to 1.01	0.68
Admission APACHE II score^b^	1.054	1.04 to 1.07	< 0.0001	1.05	1.03 to 1.07	< 0.0001
APACHE II admission category (reference level: respiratory)			< 0.0001			< 0.0001
Cardiovascular	1.39	1.11 to 1.75	0.005	1.34	1.06 to 1.69	0.015
Gastrointestinal	0.91	0.61 to 1.36	0.64	0.97	0.65 to 1.45	0.89
Neurological	0.45	0.31 to 0.66	< 0.0001	0.48	0.33 to 0.71	0.0002
Gender (female)	0.98	0.79 to 1.22	0.85	0.95	0.76 to 1.18	0.65
Total baseline SOFA^b^	1.097	1.03 to 1.17	0.005	1.002	0.94 to 1.07	0.95

APACHE, Acute Physiology and Chronic Health Evaluation; CI, confidence interval; SOFA, Sequential Organ Failure Assessment. ^a^Seventy-eight patients were excluded from the model: 61 patients with missing data on at least one of the variables included in the model, and 17 patients who have the APACHE II admission category of metabolic, of whom only one was in the statin group. ^b^Odds ratios per one-unit increase.

In univariable analysis, statin exposure was not associated with liver impairment (OR = 1.41; 95% CI = 0.89 to 2.24; *P *= 0.14; Table 4). While higher APACHE II and baseline SOFA scores and the APACHE II admission category were associated with an increased risk of liver impairment, female gender and lesser degrees of hepatic dysfunction (hepatic SOFA score = 1) appeared to be protective. After covariate adjustment, statin exposure was not associated with liver impairment (OR = 1.08; 95% CI = 0.66 to 1.77; *P *= 0.75). Increasing APACHE II score (OR = 1.05; 95% CI = 1.02 to 1.08; *P *= 0.0007) and total nonhepatic SOFA score (OR = 1.29; 95% CI = 1.11 to 1.50; *P *< 0.0009) were strongly associated with liver impairment. Patients with mild hepatic dysfunction (SOFA score = 1) had one-half the odds of developing liver impairment compared with those with no dysfunction (OR = 0.49; 95% CI = 0.30 to 0.79; *P *= 0.0032). Female patients were less likely to develop liver impairment (OR = 0.65; 95% CI = 0.42 to 99; *P *= 0.043). Treatment duration of at least 7 days was not associated with an increased risk of liver impairment (*n *= 437; OR = 0.54; 95% CI = 0.24 to 1.20; *P *= 0.13).

**Table 4:** Predictors of liver impairment and ALT > 165 IU/l occurring on or after first day of statin administration

Variable	Univariable analyses			Multivariable analyses		
		
	Odds ratio	95% CI	*P *value	Odds ratio	95% CI	*P *value
Liver impairment (*n *= 1,319)^a^						
Statin versus no statin	1.41	0.89 to 2.24	0.14	1.08	0.66 to 1.77	0.75
Age^b^	1.005	0.99 to 1.02	0.36	0.99	0.98 to 1.004	0.21
Admission APACHE II score^b^	1.06	1.03 to 1.09	< 0.0001	1.05	1.02 to 1.08	0.0007
APACHE II admission category (reference level: respiratory)			0.017			0.062
Cardiovascular	1.57	1.04 to 2.39	0.033	1.43	0.93 to 2.19	0.099
Gastrointestinal	1.46	0.76 to 2.80	0.25	1.67	0.86 to 3.25	0.13
Neurological	0.58	0.28 to 1.19	0.14	0.62	0.30 to 1.30	0.21
Gender (female)	0.62	0.41 to 0.94	0.028	0.65	0.42 to 0.99	0.043
Baseline hepatic SOFA			0.065			0.011
1 versus 0	0.58	0.36 to 0.92	0.02	0.49	0.30 to 0.79	0.0032
2 versus 0	0.85	0.49 to 1.46	0.56	0.67	0.38 to 1.18	0.17
Total baseline nonhepatic SOFA^b^	1.36	1.19 to 1.55	< 0.0001	1.29	1.11 to 1.50	0.0009
ALT (*n *= 1,229)^c^						
Statin versus no statin	2.22	1.37 to 3.60	0.001	2.25	1.32 to 3.84	0.003
Age^b^	1.00	0.98 to 1.01	0.46	0.99	0.97 to 1.002	0.091
Admission APACHE II score^b^	1.05	1.01 to 1.08	0.005	1.04	1.01 to 1.08	0.016
APACHE II admission category (reference level: respiratory)			0.054			0.12
Cardiovascular	1.47	0.89 to 2.41	0.13	1.16	0.70 to 1.94	0.57
Gastrointestinal	0.30	0.072 to 1.25	0.098	0.40	0.094 to 1.70	0.21
Neurological	1.63	0.90 to 2.94	0.11	1.80	0.97 to 3.34	0.063
Gender (female)	0.77	0.48 to 1.22	0.26	0.90	0.56 to 1.45	0.67
Baseline ALT^d^	1.14	1.08 to 1.20	< 0.0001	1.11	1.05 to 1.18	0.0001
Total baseline SOFA^b^	1.10	0.96 to 1.26	0.17	1.08	0.94 to 1.25	0.26

Predictors of liver impairment and alanine transferase (ALT) > 165 IU/l occurring on or after the first day of statin administration. APACHE, Acute Physiology and Chronic Health Evaluation; CI, confidence interval; SOFA, Sequential Organ Failure Assessment. ^a^Seventy-eight patients were excluded from the model: 61 patients with missing data on at least one of the variables included in the model, and 17 patients with the APACHE II admission category of metabolic, of whom only one was in the statin group. ^b^Odds ratios per one-unit increase. ^c^A total of 168 patients were excluded from the model: 77 patients with baseline ALT > 165 IU/l, 75 with missing data for at least one of the variables included in the model, and 16 patients with the APACHE II admission category of metabolic, of whom only one was in the statin group. ^d^Odds ratio per 10-unit increase.

In both univariable and multivariable analysis, statin exposure (adjusted OR = 2.25; 95% CI = 1.32 to 3.84; *P *= 0.003), APACHE II score (OR = 1.04; 95% CI = 1.01 to 1.08; *P *= 0.016) and admission ALT (OR = 1.11; 95% CI = 1.05 to 1.18; *P *= 0.0001) were strongly associated with a rise in ALT above 165 IU/l. Statin exposure remained associated with an ALT increase in those who received at least seven doses (*n *= 407; OR = 2.39, 95% CI = 1.25 to 4.59; *P *= 0.009).

## Discussion

The major finding from our single-centre retrospective cohort study of mechanically ventilated patients without baseline extrapulmonary organ failure is that concurrent statin therapy did not reduce the incidence of new acute organ failure. Furthermore, while statin therapy was associated with a statistically significant but clinically small rise in the ALT level, it was not associated with liver impairment as defined by changes in bilirubin. Statin-exposed patients were on average older, predominantly male and were sicker (as reflected by higher APACHE II and total SOFA scores) on admission. The overall incidence of acute organ failure in this cohort was 25.3%, took a median 3 days to develop and lasted for a median of 2 days, with no differences between statin and nonstatin groups.

The present study is the first designed specifically to investigate the effect of concurrent statin therapy on the incidence of acute organ failure in ventilated, critically ill patients. In contrast, the existing observational literature suggests that statin therapy protects against sepsis-related morbidity and mortality [11,12]. These studies have focused on pre-ICU admission chronic statin use, different populations and outcomes, and have used widely varying selection criteria. Studies exploring statin effects in the ICU have predominantly included patients with established severe sepsis.

Several smaller observational studies have investigated statin administration in the ICU and found variable effects on clinical outcomes. Fernandez and colleagues examined 438 patients at high risk of ICU-acquired infection, defined as those receiving mechanical ventilation for 96 hours [16]. Those who continued previous statin therapy while in the ICU developed statistically nonsignificantly fewer infections than statin nonusers, but were more likely to die in hospital. Schmidt and colleagues found lower mortality in 40 ICU patients with multiple organ dysfunction syndrome receiving statin therapy compared with 80 age- and sex-matched multiple organ dysfunction syndrome patients not receiving statins [17]. Dobesh and colleagues enrolled 188 patients with established severe sepsis (statin exposed, *n *= 60) and found a significantly reduced risk of hospital mortality [18]. In contrast, de Saint Martin and colleagues found no differences between statin-exposed and nonexposed patients (*n *= 921) older than 40 years of age admitted with fever in multiple outcomes (mortality, length of hospitalisation, ICU admission, and admission to convalescent homes) [19]. Finally, Kor and colleagues measured the development and progression of pulmonary and nonpulmonary organ failure for 178 patients with acute lung injury/acute respiratory distress syndrome and found no statin effects on the PaO_2_/FiO_2 _ratio and total SOFA score in univariable analyses [20]. Inferences from all these studies are subject to confounding by indication.

The present study has several strengths. First, it is the largest study specifically designed to investigate the effect of concurrent statin therapy on the incidence of acute organ failure in mechanically ventilated patients without extrapulmonary organ failure, and is the only study reporting effects on bilirubin and ALT values. Second, the study population is well-defined and clinically important, because the incidence of new acute organ failure is high and potentially preventable. Third, exposure was based on statin administration rather than prescription. Lastly, the statistical models appropriately account for repeated measures of daily assessment organ function per patient.

Our study shares several limitations of the existing observational literature. First, although we conducted careful multivariable analyses, we cannot eliminate the possibility of residual confounding. In particular, we did not know the clinical indications for statin treatment, but used the APACHE II admission category, which includes a cardiovascular category, as an adjustment variable. In contrast, information bias seems less likely since only 2.9% of eligible patients were excluded and 4.4% were excluded from regression models due to missing data. Second, we did not have data on preadmission statin treatment, leaving the possibility that a significant proportion of those patients deemed unexposed may have been prior users. During the study period our unit policy was to continue previous statin therapy, and dedicated ICU pharmacists routinely interviewed patients, relatives and the patients' general practitioners to obtain information on all chronic medications. If acute withdrawal of statin therapy is harmful, the effect of this misclassification bias would tend to diminish any findings of benefit [21]. Importantly, recently published data from a randomised controlled trial in which critically ill patients were randomised to continue or stop prior atorvastatin treatment showed no outcome differences between the exposure groups [22]. Third, we cannot confirm whether statins were actually absorbed after enteral administration. Recent data do show, however, that enteral atorvastatin is well absorbed [23]. Fourth, we were unable to study the association between statin therapy and muscle complications because creatinine kinase is not routinely collected for clinical purposes and the data were therefore not available for analyses. Fifth, we may have underestimated the association between statin administration and outcomes due to the effect of immortal time bias [24]. Finally, the study was performed using data obtained from a single academic institution and may not be generalisable to other study populations.

The reasons for the apparent failure to demonstrate a benefit are unclear, but include the potential sources of error inherent in all observational methodologies, the relatively low number of doses received (median of three), or the absence of any beneficial biological effect. Although the subgroup analyses also do not suggest a beneficial effect, the results must be interpreted carefully given that the analyses were *post hoc *and the study was not designed with these analyses in mind. Craig and colleagues, however, recently showed that treatment with simvastatin appears to be safe and may be associated with an improvement in organ dysfunction in acute lung injury [25]. Lastly, although we previously established biological plausibility and proposed biological pathways modulated by statin therapy, it is possible that these pathways are not the ones involved in the development of acute organ failure [7,9].

## Conclusions

Based on these results, concurrent statin therapy does not appear to protect against the development of new acute organ failure in critically ill, ventilated patients, but it does not appear to cause liver failure. While therapy was associated with a rise in ALT, the clinical relevance of this finding is unclear. Given the limited inferences from observational data and persistent biological rationale for the benefit of statin administration in this population at high risk of organ failure, sufficiently powered randomised controlled trials are needed.

## Key messages

• Many patients with early sepsis develop acute organ failure, and no mediator-targeting treatments able to prevent this progression are available.

• Current observational data suggest that statins may prevent sepsis-related morbidity and mortality.

• In the largest study specifically designed to test whether statins prevent the onset of new acute organ failure in ventilated ICU patients, we found no evidence of protection.

## Abbreviations

ALT: alanine transferase; APACHE: Acute Physiology and Chronic Health Evaluation; CI: confidence interval; ICU: intensive care unit; IU: international units; OR: odds ratio; PaO_2_/FiO_2_: partial pressure of arterial oxygen/inspired fraction of oxygen; SOFA: Sequential Organ Failure Assessment.

## Competing interests

The authors declare that they have no competing interests.

## Authors' contributions

MJT conceived the study and developed the protocol, participated in the data collection and analysis, and drafted the manuscript. RP analysed the data and contributed to the drafting of the manuscript. CW contributed to the study design, collected data and reviewed the manuscript. NKJA contributed to study design, data analysis, drafting of the manuscript, and revision of the manuscript for important intellectual content. SB and RB contributed to the study design, interpretation of results and appraised the manuscript for important intellectual content. All authors read and approved the final version of the manuscript.
